# Physiology

**Published:** 1860-09

**Authors:** S. Weir Mitchell

**Affiliations:** Lecturer on Physiology in the Phila. Med. Association


### PHYSIOLOGY.

BY S. WEIR MITCHELL, M.D.,

LECTURER ON PHYSIOLOGY IN THE PHILA. MED. ASSOCIATION.

### *Anatomy and Physiology; the Reproduction of Bone.*

During the last two years, a series of interesting experiments has been carried on by M. Leopold Ollier on the function of the periosteum as a producer and regenerator of osseous tissue. The subject is of some importance in a surgical as well as in a physiological point of view; and we propose to give a full abstract of M. Ollier's papers, combining with them such other notices as we have been able to find in the journals. The following are the sources from which we derive our information, the principal being the essays of M. Ollier, in *Journal de la Physiologie.*

1. *Berruti, M.* Experiments on the Transplantation of Periosteum, Giornale delle Scienze Mediche della Reale Academia de Torino, and Gazette Hebdomadaire, July 1, 1859.

2. *Flourens, M.* On the Dura Mater or Internal Periosteum of the Skull, Gaz. Méd. de Paris, August 20, 1859; on the Osteoplastic Properties of the Dura Mater, and on Cerebral Osteophytes, Gaz. Hebdom., September 9.

3. *Larghi, Dr.* Contributions to the History of Subperiosteal and Subcapsular Operations, Gaz. Méd. de Paris, January 29, February 19, May 21, July 23 and 30, August 27, and Dec. 10, 1859.

4. *Ollier, M. Leopold.* Experimental Researches on the artificial Production of Bone by Transplantation of Periosteum, and on the Regeneration of Bone after complete Resection and Removal, Journal de la Physiologie, January and April, 1859; Experimental Researches on the Grafting of Bone, *ibid.*, January, 1860; the same subjects, Gaz. Méd. de Paris, April 2 and 9, 1859; Experiments on the Transplantation of the Dura Mater, showing that this membrane may be regarded as the internal periosteum of the skull, *ibid.*, September 10, 1859, and Gaz. Hebdom., August 12; Case of Subperiosteal Resection of the Elbow-joint followed by Reproduction of the Bone, *ibid*, December 3, 1859, and Gaz. Hebdom., December 2; on the Reality of the Reproduction of Bone after Subperiosteal Resection, Gaz. Méd. de Paris, January 28, 1860; on the Transplantation of Bones taken from Animals which have been dead some time, *ibid.*, same date, and March 24.

5. *Paravicini, M.* Subperiosteal Resection and Disarticulation of the Lower Jaw, Annali Universali di Medicina, and Gaz. Méd. de Paris, July 16.

6. *Rouget, M. Charles.* The Skeleton of Vertebrate Animals in relation to the Morphology of the Locomotive Apparatus, Jour. de la Phys., January, 1860.

7. *Sédillot, M.* On Regeneration of Joints after Resection, Gaz. Méd. de Paris, November 12, 1859; on Subperiosteal Resection, *ibid.*, December 31, and Gaz. Hebdom., December 30.

Taking as a ground-work M. Ollier's papers, we find him first describing the results of his experiments on the artificial production of bone by transplantation of the periosteum.

The experiments were performed on rabbits of various ages. A marked difference was observed in the results according to the hygienic condition of the animals. In healthy rabbits, operated on in the country, union most frequently commenced at once, and in four or five days the secretion of ossifiable blastema could be felt through the skin. In animals, on the contrary, kept in Paris, in cages or boxes, death frequently followed the operation. After operation, the animals were kept in a cage during two or three days, after which they were allowed more freedom. A good supply of food is necessary, otherwise the work of reparation is arrested. In proof of this, M. Ollier removed the radius and the second metatarsal bone of a rabbit, and placed the animal for three weeks in a small, unhealthy place, with scanty food. Although the periosteum had been preserved, reparation had scarcely commenced at the end of the time. The operation was then repeated on the opposite side, and the rabbit was placed in an airy situation and well supplied with food. At the end of six weeks the bones removed by the second operation were found to be more completely restored than those which had been taken away at the first.

The experiments of M. Ollier are divisible into three series:—

First. Those in which a slip of periosteum was partially removed, but left more or less adherent to the bone; in these the slip was grafted among the muscles or under the skin, but still received some vessels from the bone.

Second. Those in which the periosteum was separated from its attachment to the bone from three to five days after the operation.

Third. Those in which a portion of the periosteum was entirely removed at once, and transplanted to another part.

1. *Transplantation of a flap of periosteum, left adhering to the bone by one end.*—The part operated on was the tibia; an incision having been made along the crest of the bone, the muscles were carefully turned aside, so as to denude the periosteum; the part to be detached was then marked out by a scalpel, and was dissected up, leaving one end attached; it was then introduced into a channel made for it under the skin or among the muscles, and the free end was fastened by a suture, to prevent it from becoming twisted. In three or four days, in young, healthy rabbits, the piece acquired a cartilaginous consistence, and sometimes became almost as thick as the tibia itself; but whether this was from infiltration from the surrounding tissues or from the blastema intended for the formation of bone, it soon diminished and gradually became more distinct and osseous. In almost all the experiments, there was a small nucleus of bone developed in the end of the periosteum beyond the suture. If the flap of periosteum escaped from the suture, it took the form of a spur, which projected more or less under the skin. In old rabbits the result was different, the wounds secreted a serous pus, and there was, at the end of a month or six weeks, little or no trace of ossification. This shows that as age advances the osteogenetic power is diminished, though not entirely abolished.

To meet the objection that periosteum merely conveyed the blastema from the bone, M. Ollier calls attention to the formation of an osseous nucleus beyond the point where the periosteum was strangled by the suture. He also having detached a flap of periosteum, twisted it several times on itself and fastened the end by suture, as in the other experiments; the result was the formation, not of a perfect ring of bone, but of a circle formed of several separate movable osseous deposits, like a string of beads.

2. *Excision of the pedicle of communication three or four days after operation.*—In these experiments the ossific process continued. In one instance the newly formed bone became reunited to the tibia, but in another, where a larger portion of the attached end was removed, the new bone was found to be joined to the tibia only by some fibrous deposit.

3. *Complete removal of a portion of periosteum and transplantation into neighboring or distant parts.*—M. Ollier dissected a piece of periosteum from the tibia of a rabbit eleven months old, leaving it attached by a few filaments of the extensor muscle of the great toe. Six weeks afterwards the piece had ossified, and was entirely independent of and movable on the tibia. In other experiments he transplanted portions of periosteum from the tibia under the skin of the groin, the back, and the popliteal space; in these cases, also, bone was formed. The new bone was not equal in size to the removed portion of periosteum from the shrinking which the latter had undergone.

The osteogenetic property does not reside alike in the periosteum of all bones. M. Ollier dissected up a portion of the pericranium of a rabbit three months old, leaving it attached at one end, and fastened it by suture under the skin so that the deep surface was turned outward. At the end of six weeks, there was but very slight trace of ossification, except along the edge of the slip where it still adhered to the bone. This result is in conformity with the demonstration of Heine, that the pericranium is not of itself sufficient for the reparation of bone.

At a meeting of the Societé de Biologie, in July, 1859, M. Ollier exhibited some specimens bearing on the question whether the dura mater performs the functions of a periosteal membrane. He transplanted portions of the dura mater of young rabbits under the skin in various parts of other animals of the same kind; at the end of five or six weeks portions of bone were found to be formed. The osteogenetic property of the dura mater he found to diminish with the age of the animal, and this, he thinks, explains why bone is so often not reproduced after the operation of trephining. Bone was not formed from the parts of the dura mater not in direct contact with the skull—the falx cerebri, for instance; pathological ossification, however, takes place very frequently in this situation. Ossification was more abundant in portions taken from the base than from the concavity of the skull.

M. Berruti has performed some experiments which confirm those of M. Ollier. In dogs he has raised flaps of periosteum, leaving them attached at one end, and has laid them with their inner surface in contact with a muscle; a plate has been formed, having all the histological characters of bone. In another instance he removed a flap of periosteum from the radius of a young dog, and fixed it to the muscle of one of the thighs of the same animal. The wound in the skin united without suppuration. On examination, after some time, the muscle was found to have a bony plate deposited in it. In another similar experiment, the transformation into bone was observed to be in progress. M. Berruti did not find any trace of cartilage in the tissue undergoing the osseous transformation.

*External characters and structure of the bone formed by transplantation of periosteum.*—The heleiotopic bone presents all the characters of normal bone. [M. Ollier uses the term heterotopique, (from *eteros*, another, and *topos*, a place,) to denote development in abnormal or extraordinary situations. We retain it in an English form, as being convenient and expressive.] Externally it is covered by periosteum; internally it contains medullary spaces, which unite in a tolerably large canal. At the periphery, there is a layer of compact tissue. When the periosteum has been allowed to remain partly connected, the heterotopic bone contains a medullary canal perfectly distinct from that of the original bone. The canal is formed gradually, by thinning of the osseous tissue, the development of vacuoles or spaces and the removal of the trabeculæ which separate them. Bones formed from transplanted periosteum are generally smaller than the piece of membrane, probably because of the necessity for the formation of a new vascular organization, and from the contraction of the piece of periosteum; but they also present a tendency to the formation of a medullary canal in their interior.

On examining thin slices of heterotopic bones under the microscope, they are found to contain osseous corpuscles, arranged in the compact substance in distinct layers, round, vascular, or Haversian canals, not however so regularly as in normal bone; but the specimens examined by M. Ollier were, it must be noticed, not more than eight or ten weeks old. The Haversian canals are generally parallel to the axis of the bone. The medullary canal contains (*a*) free nuclei, and small cells with a distinct round nucleus; (*b*) patches of numerous nuclei, generally infiltrated with fatty granulations, and containing several nuclei analogous to the free nuclei; (*c*) fat; (*d*) fibroplastic elements, and fibrils of connective tissue; (*e*) blood-vessels. The vessels enter through one or more nutritive foramina.

The heterotopic bone frequently presents a longitudinal groove on the whole length of one of its surfaces. It is always observed on the side where the edges have been turned on themselves, toward the internal surface of the periosteum, and indicates an inequality in the secretion of the blastema. The furrow is generally noticed also on bones reproduced after subperiosteal resection; it here corresponds to the incision in the membrane, through which the bone has been removed.

At their commencement, at least, the heterotopic bone is more or less adherent to the neighboring parts; the external surface of the periosteum is less distinct from the surrounding cellular tissue than the periosteum of normal bone. When developed round the leg the bone adheres to the sheaths of the muscles, so as to impede the action of these organs. At first, the tendons also are more or less completely fixed to the bone, but gradually motion is allowed, by means of a loose cellular tissue, which would perhaps at last form a serous bursa.

*Mode of development of heterotopic bones.*—From the first there is an effusion of lymph, at first serous, afterwards more consistent, infiltrating the slip of periosteum and the adjacent tissues. The periosteum then becomes turgescent and its capillaries are filled with blood; on its internal face there is produced an exudation, which at first sight resembles that furnished by the external surface and the neighboring tissues, but which is soon distinguishable by its greater density; it goes on increasing while the other becomes absorbed. In four or five days, or thereabouts, there is an accumulation of transparent or slightly yellowish-gray firm matter under the periosteum, or rather in it, (for the edges are glued together so as to form a complete sheath.) This blastema is chondroid, rather than cartilaginous. At about the seventh or eighth day, the deposition of earthy matter commences. It is not necessarily preceded by true cartilage, but sometimes there is a hard elastic substance, having the external characters of this tissue. Ossification commences at the centre, and advances rapidly to the periphery; and when the whole mass is permeated, the process goes on while the periosteum deposits new layers of blastema. Although the deposit of earthy matter is general, the bone remains flexible for some time, and might be considered as still cartilaginous until laid bare and examined, when it is found to be friable and finely granular. It gradually acquires the appearance and consistence of normal bone. The process here described indicates that the production of heterotopic bone is not necessarily preceded by the formation of cartilage; and this might be expected, inasmuch as it is not normally found under the periosteum.

By scraping the internal face of the periosteum at the commencement, a layer of blastema is detached. On placing this under the microscope, it is found to consist of a large number of nuclei, and of cells analogous to those which are found in the embryonic tissues. The free nuclei, which form the predominant element, lie in the midst of a more or less granular amorphous substance. Here and there same fusiform cells are found, as well as very fine fibres—the latter being more apparent if some force has been used in scraping the periosteum. There are also cells, some with one nucleus, others larger, with several nuclei. These large cells are often very regular in outline. The younger the animal the more abundant is the blastema. In this blastema the process of ossification goes on in the same manner as has been described, with regard to the formation of normal bone beneath the periosteum, by Virchow, Sharpey, Kölliker, Robin, and Rouget.

When cartilage is met with, it is rare to find cavities containing many cells; and there is nowhere the regular arrangement observed in ossific cartilage.

Do the heterotopic bones continue to increase in size? M. Ollier observes that, to answer this question, a period of several years would be required; but, in the mean time, reasoning from analogy, it is fair to infer that they would at least increase in thickness, like normal bones, since the periosteum exists in both cases.

*Nature of the process which takes place on the internal surface of the periosteum; immediate origin of the new bone.*—If after detaching a portion of periosteum, one-half of it is gently scraped on the internal surface, the osseous tissue will be formed from the part only which has not been so treated.

M. Ollier dissected up a flap of periosteum, leaving it attached at one end, and scraped the part nearest to its attachment to the bone. At the end of ten days this portion was merely fibrous, although traversed by numerous vessels; the part which had not been scraped presented a partly ossified deposit. Hence he concludes that neither vessels nor the external part of the periosteum are sufficient for the production of bone. The production of bone may also be prevented by touching the internal surface of the periosteum with a caustic.

In order to determine more closely the action of the subperiosteal blastema, M. Ollier proceeded to transplant it alone to distant parts. Having gently scraped some from a flap of periosteum, he inserted it under the skin of the axilla of one animal. In a week there were found small masses of a yellowish substance, having the external appearance of fat, but which, under the microscope, were found to contain nuclei of subperiosteal blastema, mixed with fragments of a granular fibroid substance, in which the presence of carbonate of lime was detected by hydrochloric acid. These masses, when treated with acid, presented elongated nuclei with more or less irregular outline.

M. Ollier makes some comments on the doctrines advanced a century ago by Duhamel, with reference to the function of the periosteum in the formation of bone. At first Duhamel regarded the bone as increased by the ossification of layers of the periosteum, but he subsequently modified this view; and in 1757, he thus expressed the analogy between the formation of wood and that of bone: "Wood increases by the addition of fine layers deposited between the wood and the bark. Bones increase in size by the formation of layers between the periosteum and the bone." He could not, M. Ollier observes, be more explicit at a period when the knowledge of the production of tissues was very imperfect.

*Transplantation of periosteum after death.*—In his experiments, M. Ollier took care to operate rapidly, lest the properties of the periosteum should be destroyed by the least amount of cooling and desiccation. He finds, however, that though rapidity in operation is most important to the production of regular and abundant ossification, the osteogenetic property is not at once lost. In January, 1859, he transplanted a piece of periosteum which had lain on his table seven or eight minutes, and had been washed with cold water; it became adherent and granules of bone were formed in it. In other cases he has succeeded with pieces which had been removed from fifteen to twenty minutes; and in periosteum removed from animals even as late as an hour and a half after death, the osteogenetic property was not entirely lost. He believes, moreover, that this limit may be carried still further under favorable conditions of temperature, etc., and according to the mode of death.

*Transplantation of the periosteum of an animal to another of a different species.*—In the experiment already referred to the periosteum was transplanted to animals of the same species; but M. Ollier has further tried the effects of introducing portions of periosteum under the skin of animals of different species from those from which the membrane was taken. He has thus experimented on the dog, cat, rabbit, guinea-pig, calf, goat, sheep, and hen, with the following general results:—1. The flap transplanted is entirely absorbed in a longer or shorter time. At first the wound heals; and for some time a small, hard nucleus is felt, which, however, gradually diminishes and disappears, so that no trace can be found on *post-mortem* examination. 2. The flap sloughs and is thrown off by suppuration; this result was most frequently observed in rabbits, on which periosteum from the dog had been engrafted. 3. The flap becomes encysted without suppuration. At first the experiment appears to have succeeded; the flap is retained in its place by plastic lymph; but this lymph soon becomes organized into a cyst, and the periosteum undergoes fatty change. Sometimes the cyst contains thickened pus. This latter result was observed chiefly in rabbits; the former in experiments with the combs of cocks. 4. The periosteum becomes adherent to the neighboring tissues, vessels are formed in it, and it continues to live as a fibrous membrane; but no bone is produced. This was observed several times in cases where periosteum was transplanted from the dog to the rabbit, and from the rabbit to the hen. 5. The periosteum not only forms a fibro-vascular adhesion to the neighboring tissues, but produces osseous tissue. This result, however, is very rare, even in animals of closely allied species.

*Modifications which bone undergoes when denuded; regeneration of the periosteum.*—It has long been known that a bone deprived of periosteum is not of necessity condemned to necrosis. Tenon proved this by experiment; and Macdonald, in 1799, showed that periosteum was reproduced after being destroyed. At a later period, this opinion has been supported by Cruveilhier and Flourens. In none of his experiments has M. Ollier observed a sequestrum at the part where the bone has been denuded. Immediate union takes place, or the wound suppurates.

In the first case the following phenomena are observed: In a short time after the bone is denuded, it becomes covered by a thin, soft, transparent layer, which soon becomes distinct from the effused plastic lymph. Toward the eighth day it resembles a coat of varnish spread over the bone. It unites with the edges of the old periosteum. At first it is without vessels; but three or four days later, these appear at the edges, being furnished by the periosteum; and in the centre, when they are supplied by the bone. The old periosteum is puffed up and thickened all around, and small tufts of vessels are seen advancing from the circumference to the centre of the denuded portion, so as to cover the varnish-like layer of blastema. In the mean time, cellular and fibrous elements are developed, and form a fibrous membrane analogous to periosteum.

To ascertain whether the new membrane thus formed is really periosteum, M. Ollier, in two instances, removed and transplanted it; but the experiment failed, probably from being performed at too early a stage (three weeks.) He subsequently performed other experiments, taking care to allow six or seven weeks for the formation of the new periosteum. In an instance related in the *Journal de la Physiologie* for July, 1859, he removed the newly-formed periosteum forty-three days after the first operation; it was thicker than the normal membrane, and was easily isolated. He then inserted it among the muscles of the leg; and at the end of twelve days, bone was found to be formed. Periosteum was again commencing to be formed in the bone which had been thus twice denuded.

M. Ollier differs from the opinion of M. Flourens, that the periosteum is capable of being reproduced indefinitely. He allows that periosteum may be removed and reproduced again and again; but this, he says, can occur only when the bone which it covered has not also been taken away.

Bone, when denuded, becomes slightly roughened while the new periosteum is being formed; it loses its whiteness and becomes reddish, and presents punctiform vascularity on its surface. When, however, the new periosteum is formed, the bone continues to grow regularly; and in rabbits, at the end of two months, it has regained its normal appearance.

When the wound is the seat of prolonged suppuration, the bone remains denuded for several days; then fleshy granulations appear on its surface and the superficial layer is gradually absorbed. When cicatrization is perfect, the surface of the bone is unequal; round the denuded part, at the limits of the old periosteum, there are projections, sometimes large; while more or less deep depressions are seen in the part which has been the seat of exfoliation. These inequalities tend to disappear gradually. While this work is going on, the bone is covered with a fibrous layer; but this new membrane is always more or less confounded with cicatrix outside it, so that it is not certain whether it is a true periosteum.—(*British Medical Journal*, May 26, 1860.)

